# Is the bacterial chromosome a mobile genetic element?

**DOI:** 10.1038/s41467-021-26758-y

**Published:** 2021-11-04

**Authors:** James P. J. Hall

**Affiliations:** Department of Evolution, Ecology and Behaviour, Institute of Infection, Veterinary, and Ecological Sciences, University of Liverpool, Liverpool, UK

**Keywords:** Bacterial genetics, Phage biology, Bacterial evolution

## Abstract

An outcome of phage infection, lateral transduction, has been shown to mobilize chromosomal genes between bacterial cells at rates that exceed those of mobile genetic elements such as plasmids. Does this mean that the bacterial chromosome should be considered a mobile genetic element?

## Lateral transduction: accelerating horizontal gene transfer

A quarter-century of bacterial genomics has shown that when it comes to genetics, the walls that divide bacteria from one another are far from solid. Microbial genes are seldom trapped within the confines of a cell, and horizontal gene transfer (HGT)—when individuals gain genes from those that are not their parents—is rampant^1^. From greenhouse-gas-consuming methanotrophs, to nitrogen-fixing symbionts, to deadly opportunistic pathogens, HGT is a fundamental process in the evolution of most bacteria and archaea. For post-genomic microbiologists, the principle of an exclusively bifurcating “tree” of life has given way to that of an (admittedly less poetic) reticulated “net”^2^. Taken to extremes, the preponderance of HGT could even imply that microbiomes are better conceptualized as collections of locally adaptive genes, rather than communities of locally adapted species^3^.

But cells *are* bound by membranes and cell walls. Specific mechanisms exist to enable genes to traverse these obstacles, or prevent genes from establishing following transfer, resulting in trends and barriers to the flow of genetic information^2^. Key players in this process are mobile genetic elements (MGEs)—entities that have adaptations for moving genes between strands of DNA and/or between individual cells. Though it is often the case that an MGE has evolved to transmit itself, sometimes MGEs facilitate the transfer of other genes. Such is the case in lateral transduction (LT), a recently characterized HGT process that is a consequence of integrative bacteriophage induction^4^. During the normal infection process, integrative phages insert themselves into a resident replicon (usually the chromosome) and replicate along with the cell, often for many generations. Induction, either stochastic or in response to an environmental stimulus, causes phage-encoded enzymes to replicate the phage genome, package copies into capsids, and lyse the cell to release infectious virus particles. Lateral transduction comes about from a slight deviation from this program. For some phages, the excisionase *xis*—responsible for extricating the phage genome from the surrounding chromosome—is only expressed late in the process. As a consequence, replication of the phage genome begins when it is still integrated, causing the surrounding chromosomal DNA to also become amplified, affecting the packaging of phage DNA into capsids. When capsid packaging occurs through the “headful” mechanism, phage proteins associate with a specific site in the phage genome to initiate packaging, and sequentially fill capsids with the downstream DNA. If the phage genome was excised and circular, this process would fill successive capsids with copies of the phage genome as it replicates, but the late expression of *xis* means that each subsequent capsid is filled with the next portion of downstream chromosomal DNA. The overall outcome is that tracts of chromosomal DNA downstream of the phage-insertion site become packaged into capsids, which are extremely efficient machines for delivering DNA into recipients. Injected into a new host, the capsid contents can integrate into the resident chromosome by recipient-encoded processes such as homologous recombination. LT thus enables chromosomal genes to transmit at very high frequencies^4^.

## The bacterial chromosome as a mobile genetic element?

In a thought-provoking article^5^, Humphrey et al. measure and review rates of LT in relation to other mechanisms by which genes are exchanged in *Staphylococcus aureus* and *Salmonella enterica*, two divergent species separated by billions of years of evolution. They conclude that LT is exceptionally powerful, because it enables large regions of DNA not directly associated with MGEs to horizontally transfer within species at rates often many times greater than that of genes carried directly by MGEs. Their findings challenge the implicit dichotomy of bacterial genome structure, in which “mobile” genetic elements carrying a genetic cargo move rapidly across a background of relatively immobile chromosomal or “core” genes. Instead, it appears that chromosomal genes located outwith conventional MGEs are regularly snatched into capsids and flung between cells, by virtue of being located in the LT “shadow” of an integrated phage. The authors^5^ ask: given the high rates of transmission of non-MGE genes in light of LT, should we consider the bacterial chromosome itself to be a mobile genetic element? Does LT demand that we reassess, or even abolish, the conceptual distinction between MGEs and the rest of the chromosome?

**Fig. 1:**
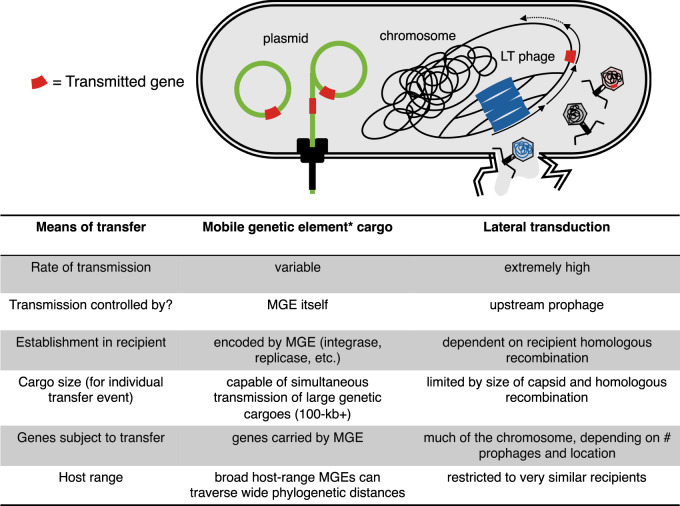
A comparison of gene transfer by lateral transduction with classical mobile genetic element cargo. *Note: as mobile genetic elements are very diverse, this column shows trends. There are likely to be exceptions.

The evidence is compelling, but I argue not. This is not to diminish the exceptionally high rates of gene transfer observed during LT, but instead to frame the question differently. The distinction that ought to be made between MGEs and chromosomal genes depends not on rates of transfer per se, but rather in terms of whether and how selection operates to shape these entities for transmission (Fig. 1). Genes exposed to HGT essentially have an alternative means of replication, placing them under multilevel selection^6^. Many MGEs, including transposons, conjugative plasmids, and bacteriophage, have evolved adaptations to exploit this opportunity, resulting in their having fitness interests distinct from other parts of the genome^7^. For example, some plasmids repress chromosomal genes for killing competing bacteria, because such competitors could become plasmid recipients^8^, while various temperate phages manipulate the behavior of their bacterial hosts to enhance phage inclusive fitness^9^. Similarly, though satellites and staphylococcal pathogenicity islands (SaPIs) are incapable of independent transmission, they too have specific adaptations to exploit other MGEs to spread to new hosts^10^. Adaptations to promote transmission can evolve because they cause the genes responsible for the adaptation to be replicated^6^. By contrast, a laterally transducing particle does not contain a copy of the phage that encodes it, nor does it necessarily encode the means of integrating its cargo into the recipient genome. Furthermore, only part of the chromosome is packaged by LT, and it is a smaller region still that is successfully integrated into the recipient, in a process that is largely beyond the control of the transferred genes. Without a genetic link between the genes for mobility, and the genes that benefit from mobility, the patterns of HGT caused by chromosomal LT are expected to be distinct from those mediated by the activity of self-interested MGEs.

Nevertheless, the exceptionally high rates of LT, and the essential differences with HGT directly mediated by MGEs, raise several exciting questions about evolution and adaptation within genomes under the influence of LT, which I discuss in the next few sections.

## Lateral transduction: *cui bono*?

First, why do phages mediate LT? Is there a benefit to the phage, such that LT could be considered a phage-level adaptation, or is LT a “spandrel” or side effect of some other phage-beneficial process?^11^ LT consumes phage resources, including replicase activity and capsids that could otherwise be used for more phage particles. Whether this cost is a significant burden is unclear—for example, what proportion of phage capsids are LT particles vs. infectious phage? However, with potentially huge effective population sizes, nonbeneficial phage traits with even very small costs are likely to be exposed to purifying selection. The fact that LT is not a universal feature of temperate phage biology indicates that in principle, phages could avoid LT and its associated costs.

So why does LT occur? There may be some mechanistic trade-off: late *xis* expression may have some advantage that outweighs the costs of LT. Another possibility is that even if LT does not benefit phages directly, it can provide indirect benefits. Many temperate phages carry accessory genes such as virulence and antibiotic-resistance factors that enhance the fitness of their bacterial hosts, with the phage benefitting in the process^9^. But though LT can effectively mobilize locally adaptive chromosomal genes, including (as Humphrey et al. show^5^) those necessary for survival in the face of environmental toxins, the phage benefits only if the recipient of the beneficial genes is also infected by the phage^12^, which may not be the case. This uncertainty weakens the power for selection to shape LT as a phage adaptation.

Alternatively, could LT be selected mainly as an adaptation at the level of the bacterial host? LT donors die in the process of releasing viral particles, and thus LT cannot benefit them directly. However, LT could be a kin-selected trait if the beneficiaries of LT are also capable of being LT donors, as is likely in spatially structured populations^6^. Alongside the adaptive opportunities offered by acquiring adaptive traits, LT enables recipients to replace deleterious alleles and remove genetic parasites such as other MGEs. Furthermore, modeling studies suggest that recombination of the sort enabled by LT is advantageous, provided the recombining population is common^13^. If LT was selected as a bacterial population-level adaptation, we might expect LT to increasingly favor chromosomal DNA packaging, and perhaps ultimately suppress phage replication altogether. Interestingly, gene-transfer agents (GTAs)—phage-like particles that are incapable of packaging their own DNA and instead package chromosomal DNA seemingly at random—appear to have reached exactly this state, and may represent one long-term evolutionary fate for those laterally transducing phages that confer a benefit at the bacterial-population level^14^.

## Genome evolution under the sway of lateral transduction

The fact that LT does not affect all genome locations equally has intriguing consequences. Humphrey et al.^5^ show how genomic islands are often located in the shadow of LT prophage, providing a compelling mechanism for the means by which such islands are mobilized. Transposons, which might benefit from the opportunity that LT offers for transmission to new hosts, could preferentially occupy regions of the genome more prone to LT. Indeed, LT may have more pervasive effects on the structure of bacterial chromosomes, if mobility is indeed determined principally by “coordinates on the chromosome”. For example, it has long been suggested that genes vary in their propensity for HGT, based on their connectivity—the degree of protein–protein interactions of their products^15^. Are LT shadows enriched in less connected, more modular genes, as a consequence? Rather than activating the entire chromosome as a mobile element, the real impact of LT may instead be to partition it, providing alternative evolutionary opportunities and fates for different resident genes. Overall, LT reinforces the disorienting perspective that a bacterial genome is perhaps better conceptualized as a loose and often temporary coalition of genes, rather than the essence of a bacterial individual.

## Some final thoughts

LT is evidentially powerful, but it is not omnipotent, and there remain barriers to gene flow (Fig. 1). Both the relatively narrow host ranges of phage and the fact that transferred DNA is thought to depend on homologous recombination for integration prevent LT across wider phylogenetic distances, homogenizing closely related genomes but placing more distantly related genomes out of reach^5^. Surface resistance to phage could thus cut a genome adrift from the flow of LT gene exchange, potentially acting as a first step in genome diversification and divergence. In addition, though much of the chromosome of each tested species could in principle be transmitted by LT, the actual amount of DNA transferred by each LT event is capped by capsid size and homologous recombination. There also remain many unknowns, not least whether LT is also undertaken by archaea. Ultimately, the evolutionary and ecological role of LT, and its impact on genome evolution, is probably quite distinct from that of HGT of MGE-encoded genes, and direct quantitative comparisons of rates fail to capture this essential qualitative difference. Nevertheless, in drawing attention to the power of LT for gene exchange in divergent species, the paper by Humphrey et al.^5^ prompts important questions about how different gene-exchange mechanisms impact genome evolution that will occupy microbiologists for some time to come.
